# 3D Digital Impression Systems Compared with Traditional Techniques in Dentistry: A Recent Data Systematic Review

**DOI:** 10.3390/ma13081982

**Published:** 2020-04-23

**Authors:** Marco Cicciù, Luca Fiorillo, Cesare D’Amico, Dario Gambino, Emanuele Mario Amantia, Luigi Laino, Salvatore Crimi, Paola Campagna, Alberto Bianchi, Alan Scott Herford, Gabriele Cervino

**Affiliations:** 1 Department of Biomedical and Dental Sciences and Morphological and Functional Imaging, Messina University, 98100 Messina, Italy; lfiorillo@unime.it (L.F.); cesaredamico89@gmail.com (C.D.); dario796@outlook.it (D.G.); emanueleamantia@gmail.com (E.M.A.); gcervino@unime.it (G.C.); 2 Multidisciplinary Department of Medical-Surgical and Odontostomatological Specialties, University of Campania “Luigi Vanvitelli”, 80100 Naples, Italy; luigi.laino@unicampania.it; 3 Department of General Surgery and Medical Surgery Specialties, University of Catania, 95100 Catania, Italy; torecrimi@gmail.com (S.C.); paolacampagna91@gmail.com (P.C.); alberto.bianchi@unict.it (A.B.); 4 Department of Oral and Maxillofacial Surgery, Loma Linda University, Loma Linda, CA 92354, USA; aherford@llu.edu

**Keywords:** dental impression technique, dental impression materials, technology, dental, diagnosis, oral, prosthodontics, digital workflow

## Abstract

The advent of new technologies in the field of medicine and dentistry is giving improvements that lead the clinicians to have materials and procedures able to improve patients’ quality of life. In dentistry, the last digital techniques offer a fully digital computerized workflow that does not include the standard multiple traditional phases. The purpose of this study is to evaluate all clinical trials and clinical randomized trials related to the digital or dental impression technique in prosthetic dentistry trying to give the readers global information about advantages and disadvantages of each procedure. Data collection was conducted in the main scientific search engines, including articles from the last 10 years, in order to obtain results that do not concern obsolete impression techniques. Elsevier, Pubmed and Embase have been screened as sources for performing the research. The results data demonstrated how the working time appears to be improved with digital workflow, but without a significant result (P = 0.72596). The papers have been selected following the Population Intervention Comparison Outcome (PICO) question, which is related to the progress on dental impression materials and technique. The comparison between dentists or practitioners with respect to classic impression procedures, and students open to new device and digital techniques seem to be the key factor on the final impression technique choice. Surely, digital techniques will end up supplanting the analogical ones altogether, improving the quality of oral rehabilitations, the economics of dental practice and also the perception by our patients.

## 1. Introduction

### 1.1. Background

Nowadays, with the advent of new technologies, the field of biomedicine, medicine and biotechnologies has also been influenced, often with improvements that affect both clinicians and patients. In dentistry, taking classical dental impressions involves the use of an anatomical or semi-arched metal or silicone/plastic impression tray spoon depending on the impression to be taken. Conventional impression techniques date back to the 1900s, and due to the development of the dental materials, new techniques have also been developed over the past few years. In the conventional impression method, the dental tray is filled with a special soft paste and inserted into the patient’s mouth so that teeth sink into the paste and is held in that position until the paste itself is completely hardened, it is few minutes.

When the dentist believes that the material has reached sufficient hardness, the spoon is removed from the mouth with extreme care to prevent the cast obtained from being altered. There are different classifications of impression materials, such as reversible and non-reversible hydrocolloids, and elastomers [1]. The former, unfortunately, do not appear to have stability over time, due to the presence of water.

At the end of the described operation, the dental technician receives the cast, which is the exact negative of the patient’s teeth and gums. To obtain the (positive) model, the plaster or the resin has to be placed inside the dental impressions and then a short wait period is required for it to be hardened.

A different recent method in relation to dental impressions is offered by the digital advent of new scanner machines. The intraoral scanner is a three-dimensional (3D) device capable of detecting dental impressions, through the first acquisition of a large number of images and then the subsequent processing using dedicated software. The possibility of acquiring an optical imprint was just hypothesized in the 70s by Dr François Duret, who is no doubt be considered the father of modern digital dentistry. In fact, he was the first to produce a dental crown by using CAD software (1983). The digital scanner, as an instrument suitable for the reproduction of real elements, must maintain a certain and infinitely reproducible coherence, with a minimum margin of error; many studies have been dedicated to developing precision in these machines, in order to identify their difference from the reality; these works have highlighted the scrupulousness of intraoral scanners, which manage to reproduce reality with a low margin of error [2,3]. One of the most obvious advantages of using such a machine is the drastic reduction of discomfort for the patients, usually reluctant to take the impression with the traditional methods (impression spoons with alginates, silicone, polyether). Furthermore, with technological progress, it has been possible to eliminate the opacification phase of the elements to be scanned, still facilitating the patient’s condition. The processing of the optical impressions obtained from the scan is much faster than with regard to the classic analog prints, with the feasibility for the medical/technical team to immediately highlight any problems and/or defects, and with the possibility of immediately showing the final results to the patient at the end of treatment (digital mock-up) [4,5]. The elimination of the development phase of the analog footprint is also an enormous advantage to digitalization of the process, thereby accelerating it significantly. The evolution of the intraoral scanner has also affected its physical appearance, making it more comfortable for its purpose, i.e., the dimensions of the apical spout have been reduced, thus, making the machine able to easily scan even the most difficult to describe dental elements with this type of system (second and third molars). The intraoral technology can be used for any type of processing, from fixed dental prosthesis to mobile, orthodontic, as well as for the identification of tooth decay. The major limit of the intraoral scanner is its high cost. Furthermore, doctors and operators have to learn quite complex skills required to master the machine with confidence [6,7,8].

### 1.2. Aim

This study investigates the clinical differences between conventional and digital impressions techniques. The principal aim is to clarify the differences between the techniques, highlighting advantages and disadvantages of each procedure.

## 2. Materials and Methods

### 2.1. Protocol and Registration

The following systematic review of the literature was registered with PROSPERO (Prospectively Registered Systematic Reviews), with protocol number 150499, dated 11/09/2019. This systematic review and meta-analysis have been conducted according to PRISMA (Transparent Reporting of Systematic Review and Meta-Analyses) protocol [9,10,11] and PICO (Population Intervention Comparison Outcome) study design [12,13,14,15,16]. Conducting a systematic review largely depends on the objective and quality of the included studies. For this reason, it may be necessary to modify the original protocol of the review during its conduct. The PRISMA Statement recognizes the dynamic nature of this process and guarantees a correct assessment of the quality of the systematic review, following a path suitable for analyzing the included studies.

### 2.2. Eligibility Criteria

Results were screened accordingly to defined eligibility criteria, inclusion and exclusion criteria were defined during the study design.

Inclusion Criteria

- Digital impression technique study
- Conventional impression technique study
- On human Study
- Randomized Controlled Trial (RCT) or Clinical Trial (CT)
- Last 10 years study.

Exclusion Criteria

- Studies involving patients with systemic disease and other pathologies
- Studies about experimental instruments
- Both accessible studies and not on English Language

### 2.3. Information Sources

The results for this systematic review have been extrapolated by the most important academic and scientific information sources as Pubmed, Embase, Elsevier, in order to obtain the highest number of results possible.

### 2.4. Search

Search terms used on information sources were: “(digital [All Fields] AND impression [All Fields] AND technique [All Fields]) AND ((Randomized Controlled Trial [ptyp] OR Clinical Trial[ptyp]) AND “loattrfull text”[sb] AND “2009/09/08”[PDat]: “2019/09/05”[PDat])”. These keywords have been elaborated by authors in order to lower risk of bias and to obtain a high number of results [17,18,19,20,21,22,23,24,25,26,27,28,29,30,31,32].

### 2.5. Study Selection

The focus question of this systematic review of PICO (Population Intervention Comparison Outcome) study design [12] is:

Are digital impression techniques more accurate and efficient in time for dental impression compared to conventional analogical techniques?

### 2.6. Data Collection Process

The authors have independently collected the data. Two independent reviewers (Luca Fiorillo and Salvatore Crimi) by two different universities collected and screened all the results. The reviewers collected data and created tables. A third expert author (Marco Cicciù) revised all the obtained data. Reviewers compared decisions and resolved differences by comparing the manuscripts. A complete independent dual revision was performed to review full-text articles.

### 2.7. Data Items

This systematic review of the literature, as already specified, was conducted in accordance with the PRISMA statement. The PICO simplification method was used to carry out the main question of this scientific article. The authors manually analyzed the data independently, and the one-way ANOVA statistical analysis was conducted on the available results. The analysis of variance (ANOVA) is a set of statistical techniques belonging to the inferential statistics where comparisons are made in the internal variability between two groups, and the variability between the groups. ANOVA is a technique developed by Fischer, used for the statistical interpretation of biological data and to test the differences between sample means. It is necessary to take into account the relative variances, in order to proceed with the analysis. The test is aimed at establishing whether two or more sample averages can be derived from populations that have the same parametric average. The analysis of variance is, therefore, used when the considered averages are greater than two.

### 2.8. Risk of Bias in Individual Studies

Authors have evaluated individual risk of bias as follow. Please see Table 1.

### 2.9. Summary Measures

According to selected studies, some measure could be compared. Main outcome of the selected results is showed in this table (Table 2 and Table 3).

### 2.10. Synthesis of Results

The results of the individual Clinical Trials and Randomized Controlled Trials have been obtained by individual authors and manually analyzed. The purpose of obtaining as many raw data as possible is to perform a meta-analysis of the results. Not all articles among the results have comparable outcomes and parameters.

### 2.11. Risk of Bias Across Studies

This type of work brings together all the studies in the literature in the last ten years demonstrating the digital instrumental investigation technique. Full text and abstract accessible articles in English have been considered. The risk of bias across studies has been evaluated according to Higgins et al. [13,14,15,16].

### 2.12. Additional Analyses

One-way ANOVA test has been conducted for Time outcome. Time was one of the outcomes evaluated and could be compared by different studies. One-Way ANOVA test considerate mean time about digital or conventional techniques for each study, where available. A mean value has been considered in studies that showed different digital techniques with different results (Table 4).

## 3. Results

### 3.1. Study Selection

**Figure 1:**
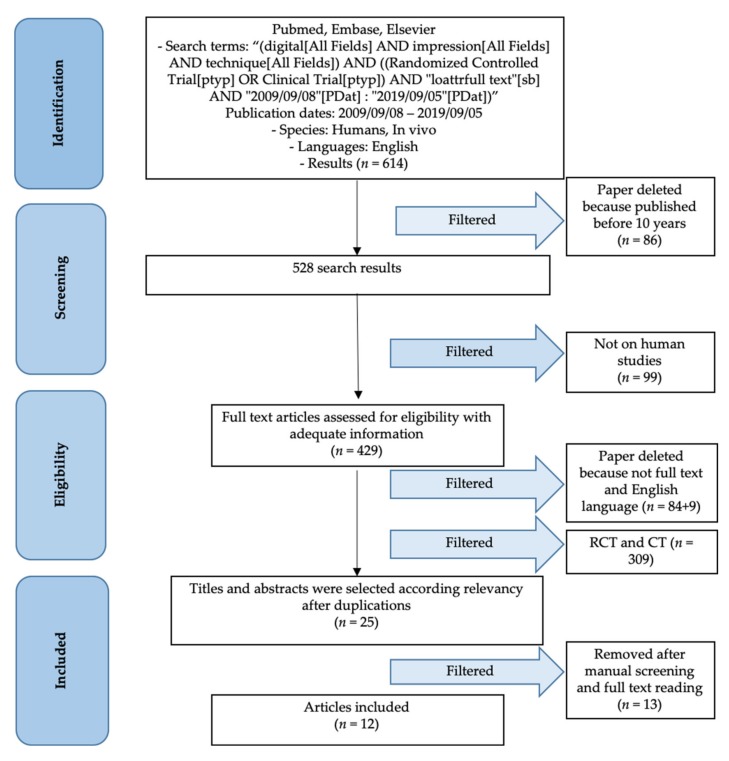
PRISMA flow chart.

**Figure 2:**
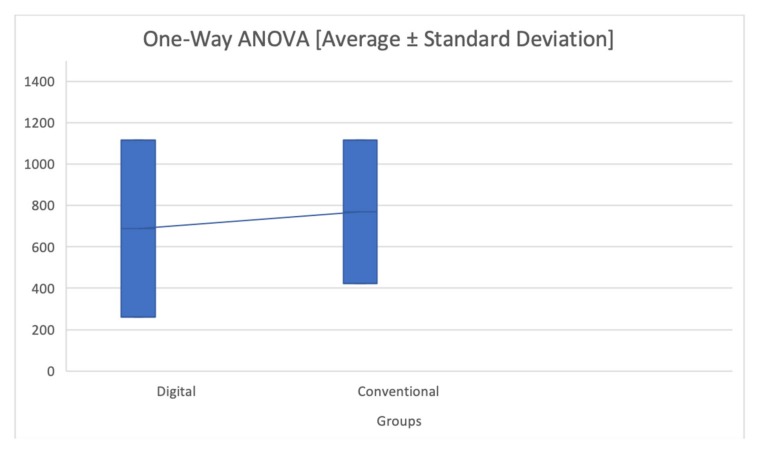
Analysis of Variance between digital and conventional impression techniques. Vertical axis: time in seconds; Horizontal axis: groups.

The results were selected based on the Materials and Method section. The first step gave a high number of results, without filters. A total of 614 papers were found and then following the first filter application (last 10 years, in order to obtain data about not obsolete instrumentation or impression techniques) the results were 528. The authors evaluated only full text article for information availability (345) on human studies (429), in English language (334). Only Randomized Clinical Trial and Clinical Trial were considered (25), and after a screening and a full text reading, only 12 articles presented sufficient information for conducting this review (Figure 1 and Figure 2).

### 3.2. Study Characteristics

Single study features have been evaluated and showed in Table 3.

### 3.3. Results of Individual Studies

Two papers recently published by Cave and Chandran have been evaluated as pertinent, but not included in the review cause not RCT [17,18].

Zitzmann et al. [19] evaluated the differences between digital and conventional techniques in VAS questionnaire results completed by dental students. The authors evaluated the TRIOS Pod system (3Shape, Copenhagen, Denmark) quotient too, and it indicated that dental student used time in digital techniques more efficiently. The majority of students perceived Intra Oral Scanner IOS as easier than the conventional technique. Most (72%) preferred the digital approach using IOS to take the implant impression to the conventional method (12%) or had no preference (12%). Zeltner et al. [20] investigated the differences in monolithic lithium disilicate crown fabrication on the same abutment with different workflows, digital or conventional. Some authors evaluated laboratory centralized milling techniques versus chairside milling techniques too. The differences between the treatment modalities (Lava, iTero, Cerec inLab, and Cerec) were not statistically significant (*p* > 0.05). Sailer et al. [21] evaluated differences between digital workflows (Lava C.O.S.; 3M [Lava], iTero; Align Technology Inc [iTero], Cerec Bluecam; Dentsply Sirona [Cerec]) and conventional techniques (Permadyne, 3M). Scan time has been evaluated with significant differences on 2 digital techniques on 3 vs. Conventional techniques. Participants preferred conventional techniques and they preferred digital method without powdering. The total time for the complete-arch impressions, including the preparation (powdering) and the occlusal registration, was shorter for the conventional impression than for the digital scans. Cappare et al. [22] evaluated differences on full arch scans with both conventional and digital techniques, differences that were not statistically significant (*p* > 0.05) in marginal bone loss were found between control and test groups. Significantly less time was spent to perform digital impression procedure (*p* < 0.05). Digital workflows needed less time once again. Sakornwimon et al. [23] evaluated marginal gap and patient’s preferences between conventional impressions (Polyvinyl siloxane) and digital scans. Crowns were evaluated intraorally through a blinded examination and a stereomicroscope; this evaluation reported no significant discrepancies as opposed to VAS results where patients. Visual analog scale scores for digital impressions were statistically significantly higher than those for PVS impressions in every topic (*p* < 0.05). Joda et al. [24] in a randomized controlled trial evaluated time, difficulty, and operator’s preference in using digital versus conventional impression techniques. Working time showed significant differences between the two groups. Difficulty and applicability of IOS was perceived more favorable compared to conventional impressions, and effectiveness of IOS was rated better by the majority of students (88%) and dentists (64%). While 76% of the students preferred IOS, 48% of the dentists were favoring conventional impressions, and 26% each IOS and either technique. Another crossover study of Joda et al. [25] evaluated outcome differences on digital intraoral scanning and polyether impressions. They assessed patients’ perception and satisfaction with a VAS questionnaire. Clinical time was recorded by an operator too. All patients would prefer the digital workflow if they could choose between the two techniques in the future. Gjelvold et al. evaluated differences on time, clinical condition and dentist and patient’s satisfaction (VAS) between digital and conventional impression techniques. The results of this study demonstrated that the digital technique was more efficient and convenient than the conventional impression technique (14:33 ± 5:27, and 20:42 ± 5:42, respectively (*p* < 0.0001)) [31]. Gherlone et al. [26] randomly selected patients who underwent full-arch immediate-load rehabilitation. They evaluated time and accuracy of digital and conventional rehabilitation. The digital impression procedure required significantly less time than the conventional procedure (*p* < 0.001). Benic et al. [27] evaluated time, patients’ discomfort and operator difficulty between 4 different techniques, 3 digital and 1 conventional with polyvinyl siloxane. Their results showed that there were no statistical differences between both, digital or conventional techniques. The total working time for the conventional impression was significantly lower than that for Lava and Cerec. With regard to the working time without powdering, the differences between the methods were not statistically significant. Boeddinghaus et al. [28] evaluated three differences on 3 intraoral scanners (Sirona CEREC AC Omnicam (OCam), Heraeus Cara TRIOS and 3M Lava True Definition (TDef)) and a conventional impression model (EXA’lence, GC, Tokyo, Japan). The authors evaluated fitting and marginal gap. Yuzbasioglu et al. [32] evaluated differences in time spending and in patient’s satisfaction with two different impression methods techniques. Conventional impressions were taken with a polyether impression material (Impregum, 3 M ESPE), and bite registrations were made with polysiloxane bite registration material (Futar D, Kettenbach). Digital impressions and bite scans were performed using an intra-oral scanner (CEREC Omnicam, Sirona). Time was shorter for digital impressions technique and patients stated that digital impressions were more comfortable than conventional techniques. [29,30].

### 3.4. Synthesis of Results

A synthesis of results has been provided in detailed form in Table 5.

### 3.5. Additional Analysis

**Figure 3:**
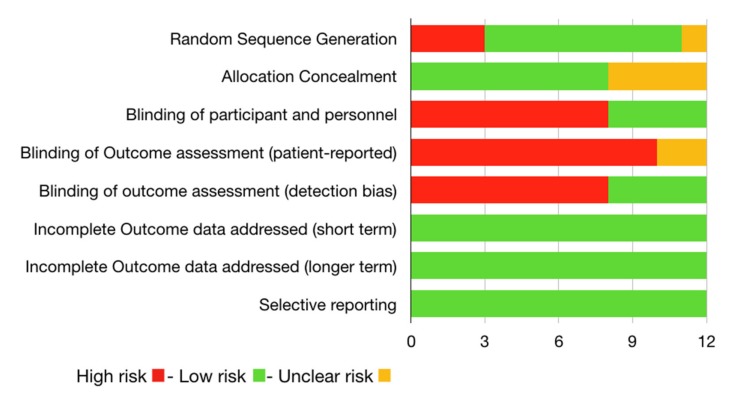
Risk of Bias according to Cochrane reviews.

According to results is possible to perform an analysis of variance about 2 different group, the considered outcome is time (Table 6 and Figure 3).

According to ANOVA test P = 0.72596.

## 4. Discussion

### 4.1. Summary of Evidence

It was possible to conduct an analysis of the treated topic, once the individual reviewer results and the conclusions of the investigated articles were extrapolated. In this section, evaluating the synthesis of the individual articles conclusions, benefits or disservices of each methodic can be summarized as follows.

Recently, Cave and Keys [17] performed a systemic review about the working time of the two impressions technique. They concluded that the digital impression technique in reducing anxiety and nausea could be considered more comfortable for the patients than a conventional impression technique. However, the topic is still highly debated in the recent literature and Chandran et al. [18] explained how the digital impressions are superior to a conventional one, without any statistically significant differences, based on assessment of accuracy, patient preference and operator preference.

Iin a RCT, Zitzmann et al. [19] Analyzed both digital and conventional using difficulty on different impression techniques. No experienced dental student found a digital tool easier than conventional impression techniques. According to Zeltner et al. [20], no significant differences were found between conventional or digital workflow in prosthodontic. Authors showed how a conventional workflow can facilitate the better manufacture of occlusal regions. Moreover, centralized milling production provided better results than chairside milling. Sailer et al. [21] in their RCT showed how digital techniques could improve chair time and how participants prefer no powder-need digital techniques for digital scans. Cappare et al. [22] evaluated how digital workflows provide accuracy and predictability. It is a reliable alternative for full arch rehabilitations with a marginal fit precision. Sakornwimon et al. [23] found that conventional and digital techniques present no differences on crowns marginal gap but patients’ satisfaction is higher with the “digital way”. Joda et al. [24] demonstrated, on a dentist and dental students’ group, how digital scanning is more efficient than conventional techniques for single implant or single quadrant impression. Also, they demonstrated a high level of acceptance by operators. Joda et Bragger [25] showed how, based on their findings, that patients preferred digital technique, particularly because of their efficiency in terms of time. Gjelvold et al. [31] concluded that the digital technique was more efficient and convenient than an analogical, conventional one. According to Gherlone et al. [26], it is possible to realize full-arch rehabilitation, with a satisfactory accuracy way, using digital instruments. Benic et al. [27] demonstrated how a conventional impression technique was more time-effective than digital, and no statistical differences were found with respect to patient discomfort. Boeddinghaus et al. [28] concluded that the digital intraoral impression could be considered a valid alternative to conventional one. Yilmax [29] in his research documented the “time” perception of the patients. The digital advent in the field of dental impression technique reduces the number of appointments and allows the formation of a soft tissue emergence profile, similar to that of the definitive crown.

A different point of view is underlined by Runkel et al. [30]. In a paper published in 2019 authors underlined that despite the rapid advancement of the computer-aided technology for dental therapy purposes, the implementation of this technique is not as fast as its technical development.

Yuzbasioglu et al. [32] demonstrated how digital methods for impressions in dentistry could be more time-efficient and preferred by patients. Some studies, therefore, consider the digital impression as optimal with regard to the economy of the time and therefore financial of the medical office. However, some studies, are inconsistent in this topic and, as can be seen, it is not a significant parameter.

Some studies in the literature report the problem of impression infection management, and the management of the latter over time, in the dental laboratory [32,33,34,35], the impression material stability during time [34], or material working phase and mixing issues [36,37,38]. This is an issue that does not exist in the case of optical impressions. As far as quality is concerned, the latter did not show statistically significant parameters. Digital equipment is starting to be used in the medical field, and above all in the dental field, it is now possible to have a completely digital workflow [4,8,39,40,41].

**Figure 4:**
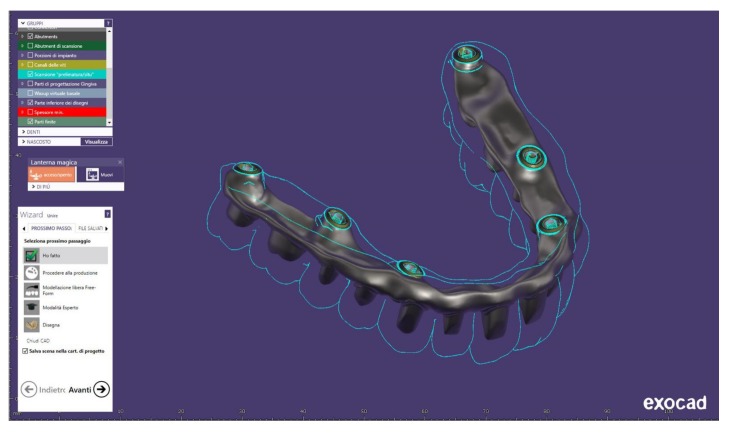
Sample of computers planning and realization of prosthodontics structure before starting the treatment over patients.

**Figure 5:**
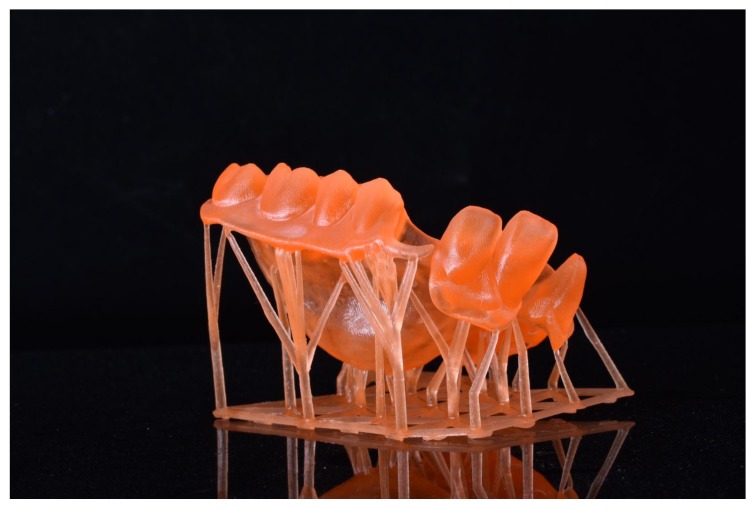
Sample of new devices like 3D printing for having dental threedimensional model.

Ortensi et al. recently demonstrated how the application of new materials and digital techniques must guarantee a predictability of the final goal from the beginning to the end of treatment. The possibility of showing the patients the planning treatment as well as the avoiding analogue impression technique is highly appreciated by the patients (Figure 4 and Figure 5) [42,43,44,45].

A, digital diagnosis, and therapeutic programming, with a digital plane preview, should be the future for clinicians and prosthodontics practitioners [46,47]. The traditional impression technique is based on a copy of the oral situation, with acquisition materials and subsequent casting in plaster. This working method has spread in clinical practice; however, the impression materials tend to contract in size due to the chemical reaction of the material. Instead, the plaster, used later, will show a dimensional expansion. It should be noted that the impression procedure is at the origin of the manufacture of the product, and therefore, potential errors introduced in this phase will affect the rest of the work. In the case of implant prostheses, a failure to adapt the scaffolding will generate stress on the implants, which will affect the bone interface, causing failure in some cases. Prosthetic complications, such as loosening of the screw or its fracture, could also be related to inadequate insertion of the prosthesis. However, no technique has proved to be effective. Impressions on implants have shown good accuracy. With an impression system, the data through the intraoral scanner could be transmitted through files to the laboratory for the manufacture of a definitive prosthesis. It is also known that implants, in response to bone compression, show only a range of motion of 3–5 µm in the axial direction and 10–50 µm in the horizontal direction. An intraoral scanner could overcome some errors associated with taking the traditional impression and in production, such as the fact that it communicates with the laboratory directly through a virtual world avoiding errors in preserving the impression. In the literature, there are reports regarding the digital impression technique on dental implants, but most deal with fabrications of customized anatomical abutments and zirconia prostheses. All definitive prostheses, with the different cemented, screwed methods, require accuracy in the bar-implant connection. The scanner copies the implant fixture exactly in the mouth like traditional impressions. Once the image is captured and registered by an intraoral scanner, the CAD software through algorithms could precisely position the implant in the virtual model. In addition, the new technological developments of the optical impression provide the digital creation of a model through analogues, as the traditional laboratory technique requires. Registration errors, however minimal, occur during the acquisition procedures, arising from the length of the arch. When comparing intraoral scanners in whole arch acquisition procedures, the acquisition width should be considered to consider the errors that can be encountered. Once the scan has taken place and the data has been acquired, the software processes every single data to create a virtual 3D model, then the CAD builds the resin model from the collected data. The lack of homogeneity in the results between the cited studies indicates that it is not possible to determine a conclusion about whether the working time appears to improve with digital workflow. Indeed, a significant change is the introduction of digital technology into dental practice. “Digital Dentistry” is becoming more prevalent each year. Recently, digital impression techniques with three-dimensional (3D) intra-oral scanners have been attracting attention gaining in popularity around the world. These intra-oral scanners capture digital images of the dental arches and record occlusal relationships, which could directly be used for computer aided design (CAD) and manufacture (CAM) of a dental prosthesis. Intra-oral scanners have the potential to replace conventional impression materials for several reasons. However, accuracy and precision of the impression may be influenced by various clinical factors, such as the difference in the operator’s skill or the patients’ condition, which could only be evaluated by in vivo studies. To date, only a small number of in vivo studies, investigating the accuracy of this impression technique has been reported. Accuracy could only be evaluated in comparison, preferably with a gold standard; which is not easy to establish in the oral cavity. Regarding precision, there is only a limited number of in vivo studies in the literature [48,49,50,51].

### 4.2. Limitations

The main limitation of the study is the low number of works evaluated, despite the fact that almost all of them have agreed results. The studies included in this review, unfortunately, presented data not comparable to each other. Therefore, it was not possible to conduct a meta-analysis of the literature. The only comparable numerical data was that of time. Certainly, further studies will be necessary, and useful to obtain more precise information about these techniques, which over time, will replace the analog ones.

## 5. Conclusions

According to the obtained results in this systematic review, it is certainly possible to say that digital techniques represent a valid alternative in the field of dentistry. The optical impression system compared to the analogue one with the impression materials has a comparable result. Moreover, it is necessary to remember how dentists appeared more distrustful in difficulty, compared with dentistry students. Furthermore, patients have a better perception of the use of digital rather than conventional impressions. The total work time for the impression taking would appear to be lower with digital techniques, but despite this, the data is still not significant. The authors recommend the use of intraoral scanners, which from the formation of a virtual image creates an accurate physical model that gives efficiency to the dental structure and makes the work lighter. This improved way of working should benefit the dentist, the laboratory and the patient.

## Figures and Tables

**Table 1:** Individual Risk of Bias Table.

Author and Year	Risk of Bias			
	Unclear	Low	Moderate	High
Zitzmann et al. 2017 [19]		x		
Zeltner et al. 2017 [20]			x	
Sailer et al. 2019 [21]			x	
Capparè et al. 2019 [22]			x	
Sakornwimon et al. 2017 [23]		x		
Joda et al. 2017 [24]			x	
Joda et al. 2016 [25]		x		
Gherlone et al. 2016 [26]		x		
Benic et al. 2016 [27]		x		
Boeddinghaus et al. 2015 [28]			x	
Gjelvold et al. 2016 [31]		x		
Yuzbasioglu et al. 2014 [32]			x	

**Table 2:** Risk of bias according to Cochrane reviews.

Author and Year	Entry	Risk of Bias	Support for Judgement
Zitzmann et al. 2017 [19]	Random Sequence Generation (selection bias)	High risk.	“Fifty undergraduate dental students with no clinical experience at the School of Dental Medicine, University of Basel, Switzerland were included in the study.” (Maybe no randomly)
	Allocation Concealment (selection bias)	Low risk.	“Randomly divided”
	Blinding of participant and personnel (performance bias)	High risk	(cannot be conducted on digital vs. conventional impression techniques)
	Blinding of outcome assessment (detection bias) (patient-reported outcomes)	High risk	(cannot be conducted on digital vs. conventional impression techniques)
	Blinding of outcome assessment (detection bias) (Mortality)	High risk	(cannot be conducted on digital vs. conventional impression techniques)
	Incomplete outcome data addressed (attrition bias) (Short-term outcomes (2–6 weeks))	Low risk	(outcomes by all participants)
	Incomplete outcome data addressed (attrition bias) (Longer-term outcomes (>6 weeks))	Low risk	(outcomes by all participants)
	Selective reporting (reporting bias)	Low risk	Statistics has been performed on all outcomes
Zeltner et al. 2017 [20]	Random Sequence Generation (selection bias)	Low risk	Random selection of participants
	Allocation Concealment (selection bias)	Low risk	“The sequence of the crown assessment was randomly allocated according to a computer-generated list.”
	Blinding of participant and personnel (performance bias)	Low risk	“To eliminate operator bias, the investigators generated and evaluated the replicas without being able to distinguish among the crowns under investigation.”
	Blinding of outcome assessment (detection bias) (patient-reported outcomes)	High risk	Not applicable
	Blinding of outcome assessment (detection bias) (Mortality)	Low risk	Blinded outcome assessment
	Incomplete outcome data addressed (attrition bias) (Short-term outcomes (2–6 weeks))	Low risk	Complete data
	Incomplete outcome data addressed (attrition bias) (Longer-term outcomes (>6 weeks))	Low risk	Complete data
	Selective reporting (reporting bias)	Low risk	Complete data
Sailer et al. 2019 [21]	Random Sequence Generation (selection bias)	Low risk	10 participants in need of a tooth supported 3-unit fixed denture included
	Allocation Concealment (selection bias)	Low risk.	“Software (www.randomizer.org) was used to create a computer-generated list of 10 sequences of the 4 tested scanning or impression procedures”
	Blinding of participant and personnel (performance bias)	High risk	“The same clinician carried out all the scanning and impression making on the assigned participants.”
	Blinding of outcome assessment (detection bias) (patient-reported outcomes)	High risk	Not specified (maybe no blinding)
	Blinding of outcome assessment (detection bias) (Mortality)	High risk.	Not specified (maybe no blinding)
	Incomplete outcome data addressed (attrition bias) (Short-term outcomes (2–6 weeks))	Low risk	Complete data
	Incomplete outcome data addressed (attrition bias) (Longer-term outcomes (>6 weeks))	Low risk	Complete data
	Selective reporting (reporting bias)	Low risk	No selective reporting
Capparè et al. 2019 [20]	Random Sequence Generation (selection bias)	Low risk	“patients were randomly selected for this clinical study”
	Allocation Concealment (selection bias)	Low risk	Patients have been scheduled randomly into control (conventional impression group, CIG) and test (digital impression group, DIG) groups respectively for a fully conventional workflow and a fully digital workflow.
	Blinding of participant and personnel (performance bias)	Low risk	“Randomization processes occurred by lots in closed envelopes and were performed by a blinded operator”
	Blinding of outcome assessment (detection bias) (patient-reported outcomes)	High risk	(cannot be conducted on digital vs. conventional impression techniques)
	Blinding of outcome assessment (detection bias) (Mortality)	High risk	(cannot be conducted on digital vs. conventional impression techniques)
	Incomplete outcome data addressed (attrition bias) (Short-term outcomes (2–6 weeks))	Low risk	Follow up to 24 months
	Incomplete outcome data addressed (attrition bias) (Longer-term outcomes (>6 weeks))	Low risk	Follow up to 24 months
	Selective reporting (reporting bias)	Low risk	No selective reporting
Sakornwimon et al. 2017 [23]	Random Sequence Generation (selection bias)	High risk	Not random selection
	Allocation Concealment (selection bias)	Unclear risk	Not specified, maybe not
	Blinding of participant and personnel (performance bias)	Low risk	Blinded operator
	Blinding of outcome assessment (detection bias) (patient-reported outcomes)	Unclear risk	No patient reported outcomes
	Blinding of outcome assessment (detection bias) (Mortality)	Low risk	Blinded
	Incomplete outcome data addressed (attrition bias) (Short-term outcomes (2–6 weeks))	Low risk	Complete data
	Incomplete outcome data addressed (attrition bias) (Longer-term outcomes (>6 weeks))	Low risk	Complete data
	Selective reporting (reporting bias)	Low risk	Complete data
Joda et al. 2017 [24]	Random Sequence Generation (selection bias)	Low risk	Randomly selected
	Allocation Concealment (selection bias)	Unclear risk	Not specified
	Blinding of participant and personnel (performance bias)	High risk	(cannot be conducted on digital vs. conventional impression techniques)
	Blinding of outcome assessment (detection bias) (patient-reported outcomes)	High risk	No blinding
	Blinding of outcome assessment (detection bias) (Mortality)	High risk	No blinding
	Incomplete outcome data addressed (attrition bias) (Short-term outcomes (2–6 weeks))	Low risk	Complete data
	Incomplete outcome data addressed (attrition bias) (Longer-term outcomes (>6 weeks))	Low risk	Complete data
	Selective reporting (reporting bias)	Low risk	Complete data
Joda et al. 2016 [25]	Random Sequence Generation (selection bias)	Low risk	Random selection
	Allocation Concealment (selection bias)	Low risk	“Random allocation”
	Blinding of participant and personnel (performance bias)	High risk	(cannot be conducted on digital vs. conventional impression techniques)
	Blinding of outcome assessment (detection bias) (patient-reported outcomes)	High risk	(cannot be conducted on digital vs. conventional impression techniques)
	Blinding of outcome assessment (detection bias) (Mortality)	High risk	(cannot be conducted on digital vs. conventional impression techniques)
	Incomplete outcome data addressed (attrition bias) (Short-term outcomes (2–6 weeks))	Low risk	Complete data
	Incomplete outcome data addressed (attrition bias) (Longer-term outcomes (>6 weeks))	Low risk	Complete data
	Selective reporting (reporting bias)	Low risk	Complete data
Gjelvold et al. 2016 [31]	Random Sequence Generation (selection bias)	Low risk	Randomly selection
	Allocation Concealment (selection bias)	Low risk	Randomly allocation
	Blinding of participant and personnel (performance bias)	High risk	(cannot be conducted on digital vs. conventional impression techniques)
	Blinding of outcome assessment (detection bias) (patient-reported outcomes)	Unclear risk	Not applied
	Blinding of outcome assessment (detection bias) (Mortality)	Low risk	“This dentist was not present at the dental office when the impressions were taken”
	Incomplete outcome data addressed (attrition bias) (Short-term outcomes (2–6 weeks))	Low risk	Complete data
	Incomplete outcome data addressed (attrition bias) (Longer-term outcomes (>6 weeks))	Low risk	Complete data
	Selective reporting (reporting bias)	Low risk	Complete data
Gherlone et al. 2016 [26]	Random Sequence Generation (selection bias)	Low risk	Randomly selected
	Allocation Concealment (selection bias)	Low risk	Randomly allocated
	Blinding of participant and personnel (performance bias)	High risk	(cannot be conducted on digital vs. conventional impression techniques)
	Blinding of outcome assessment (detection bias) (patient-reported outcomes)	High risk	Not performed
	Blinding of outcome assessment (detection bias) (Mortality)	High risk	Not performed
	Incomplete outcome data addressed (attrition bias) (Short-term outcomes (2–6 weeks))	Low risk	Complete data
	Incomplete outcome data addressed (attrition bias) (Longer-term outcomes (>6 weeks))	Low risk	Complete data
	Selective reporting (reporting bias)	Low risk	Complete data
Benic et al. 2016 [27]	Random Sequence Generation (selection bias)	Low risk	Randomly selected
	Allocation Concealment (selection bias)	Low risk	Randomly allocated
	Blinding of participant and personnel (performance bias)	Low risk	Blinded personnel
	Blinding of outcome assessment (detection bias) (patient-reported outcomes)	High risk	Not applicable
	Blinding of outcome assessment (detection bias) (Mortality)	Low risk	“The impression sequences were concealed by means of sealed envelopes until the time of the clinical procedure that required the tooth impression.”
	Incomplete outcome data addressed (attrition bias) (Short-term outcomes (2–6 weeks))	Low risk	Complete data
	Incomplete outcome data addressed (attrition bias) (Longer-term outcomes (>6 weeks))	Low risk	Complete data
	Selective reporting (reporting bias)	Low risk	Complete data
Boeddinghaus et al. 2015 [28]	Random Sequence Generation (selection bias)	High risk	Not highlighted
	Allocation Concealment (selection bias)	Unclear risk	Not specified
	Blinding of participant and personnel (performance bias)	High risk	(cannot be conducted on digital vs. conventional impression techniques)
	Blinding of outcome assessment (detection bias) (patient-reported outcomes)	High risk	High risk
	Blinding of outcome assessment (detection bias) (Mortality)	High risk	Random application only of digital technique
	Incomplete outcome data addressed (attrition bias) (Short-term outcomes (2–6 weeks))	Low risk	Complete data
	Incomplete outcome data addressed (attrition bias) (Longer-term outcomes (>6 weeks))	Low risk	Complete data
	Selective reporting (reporting bias)	Low risk	Complete data
Yuzbasioglu et al. 2014 [32]	Random Sequence Generation (selection bias)	Unclear risk	Not specified
	Allocation Concealment (selection bias)	Unclear risk	Not specified
	Blinding of participant and personnel (performance bias)	High risk	(cannot be conducted on digital vs. conventional impression techniques)
	Blinding of outcome assessment (detection bias) (patient-reported outcomes)	High risk	Not specified
	Blinding of outcome assessment (detection bias) (Mortality)	High risk	Not specified
	Incomplete outcome data addressed (attrition bias) (Short-term outcomes (2–6 weeks))	Low risk	Complete data
	Incomplete outcome data addressed (attrition bias) (Longer-term outcomes (>6 weeks))	Low risk	Complete data
	Selective reporting (reporting bias)	Low risk	Complete data

**Table 3:** Outcomes by the results.

Outcomes
Visual Analog Scales (VAS), Student preference, Time, Occlusal gap, Marginal gap, Discrepancy Shoulder, framework/implant connection (Radiographic evaluation), voids at the bar/implant, bone level, operator preference, discomfort, accuracy, Patients’ perceptions (VAS), Operator difficulty (VAS).

**Table 4:** Study characteristics.

Author and Year	Outcomes
Zitzmann et al. 2017 [19]	Visual Analog Scales (VAS), Student preference, Time.
Zeltner et al. 2017 [20]	Marginal gap, Discrepancy Shoulder, Chairside vs. Centralized techniques, Occlusal gap
Sailer et al. 2019 [21]	Time, Occlusal registration, VAS
Cappare et al. 2019 [22]	Time, framework/implant connection (Radiographic evaluation), voids at the bar/implant, bone level.
Sakornwimon et al. 2017 [23]	Marginal fit, patient’s preferences (VAS)
Joda et al. 2017 [24]	Time, difficulty, operator preference
Joda et al. 2016 [25]	Patients’ satisfaction (VAS), Time
Gjelvold et al. 2016 [31]	Time; difficulty; discomfort; occlusal gap.
Gherlone et al. 2016 [26]	Time, Accuracy
Benic et al. 2017 [27]	Time, Patients’ perceptions (VAS), Operator difficulty (VAS).
Boeddinghaus et al. 2015 [28]	Crown fit and marginal gap
Yuzbasioglu et al. 2014 [32]	Time, Patient’s satisfaction

**Table 5:** Study characteristics.

Author and Year	Outcomes
Zitzmann et al. 2017 [19]	Visual Analog Scales (VAS), Student preference, Time.
Zeltner et al. 2017 [20]	Marginal gap, Discrepancy Shoulder, Chairside vs. Centralized techniques, Occlusal gap
Sailer et al. 2019 [21]	Time, Occlusal registration, VAS
Cappare et al. 2019 [22]	Time, framework/implant connection (Radiographic evaluation), voids at the bar/implant, bone level.
Sakornwimon et al. 2017 [23]	Marginal fit, patient’s preferences (VAS)
Joda et al. 2017 [24]	Time, difficulty, operator preference
Joda et al. 2016 [25]	Patients’ satisfaction (VAS), Time
Gjelvold et al. 2016 [31]	Time; difficulty; discomfort; occlusal gap.
Gherlone et al. 2016 [26]	Time, Accuracy
Benic et al. 2017 [27]	Time, Patients’ perceptions (VAS), Operator difficulty (VAS).
Boeddinghaus et al. 2015 [28]	Crown fit and marginal gap
Yuzbasioglu et al. 2014 [32]	Time, Patient’s satisfaction

**Table 6:** Analysis of Variance.

Data Summary about Time				
Groups	Number of Measures	Mean	Standard Deviation	Standard Error
Digital	6	689.15	428.5407	174.951
Conventional	6	770.2833	346.7292	141.5516
